# An 8.22 Mb Assembly and Annotation of the Alpaca (*Vicugna pacos*) Y Chromosome

**DOI:** 10.3390/genes12010105

**Published:** 2021-01-16

**Authors:** Matthew J. Jevit, Brian W. Davis, Caitlin Castaneda, Andrew Hillhouse, Rytis Juras, Vladimir A. Trifonov, Ahmed Tibary, Jorge C. Pereira, Malcolm A. Ferguson-Smith, Terje Raudsepp

**Affiliations:** 1 Department of Veterinary Integrative Biosciences, College of Veterinary Medicine and Biomedical Sciences, Texas A&M University, College Station, TX 77843-4458, USA; mjevit@cvm.tamu.edu (M.J.J.); bdavis@cvm.tamu.edu (B.W.D.); ccastaneda@cvm.tamu.edu (C.C.); rjuras@cvm.tamu.edu (R.J.); 2 Molecular Genomics Workplace, Institute for Genome Sciences and Society, Texas A&M University, College Station, TX 77843-4458, USA; hillhouse@tamu.edu; 3 Laboratory of Comparative Genomics, Institute of Molecular and Cellular Biology, 630090 Novosibirsk, Russia; vlad@mcb.nsc.ru; 4 Department of Veterinary Clinical Sciences, College of Veterinary Medicine, Washington State University, Pullman, WA 99164-6610, USA; tibary@vetmed.wsu.edu; 5 Department of Veterinary Medicine, University of Cambridge, Cambridge CB3 0ES, UK; jorgecpereira599@gmail.com (J.C.P.); maf12@cam.ac.uk (M.A.F.-S.)

**Keywords:** camelid, alpaca, Y chromosome, de novo assembly, cytogenetics, pseudoautosomal, comparative, PacBio, Illumina

## Abstract

The unique evolutionary dynamics and complex structure make the Y chromosome the most diverse and least understood region in the mammalian genome, despite its undisputable role in sex determination, development, and male fertility. Here we present the first contig-level annotated draft assembly for the alpaca (*Vicugna pacos*) Y chromosome based on hybrid assembly of short- and long-read sequence data of flow-sorted Y. The latter was also used for cDNA selection providing Y-enriched testis transcriptome for annotation. The final assembly of 8.22 Mb comprised 4.5 Mb of male specific Y (MSY) and 3.7 Mb of the pseudoautosomal region. In MSY, we annotated 15 X-degenerate genes and two novel transcripts, but no transposed sequences. Two MSY genes, *HSFY* and *RBMY,* are multicopy. The pseudoautosomal boundary is located between *SHROOM2* and *HSFY.* Comparative analysis shows that the small and cytogenetically distinct alpaca Y shares most of MSY sequences with the larger dromedary and Bactrian camel Y chromosomes. Most of alpaca X-degenerate genes are also shared with other mammalian MSYs, though *WWC3Y* is Y-specific only in alpaca/camels and the horse. The partial alpaca Y assembly is a starting point for further expansion and will have applications in the study of camelid populations and male biology.

## 1. Introduction

Mammalian sex chromosomes, the X and the Y, evolved from a pair of autosomes, and diverged from each other approximately 180 million years ago by gradual suppression of crossing-over due to inversions and other mutations accumulating in the Y chromosome [1,2,3,4,5,6]. As a result, the majority of the Y chromosome is non-recombining, except for the pseudoautosomal region (PAR) [7,8]. Hemizygosity and the lack of recombination of the male specific Y (MSY) provide a unique environment for the expression of Y chromosome genes [9,10,11] and facilitate the accumulation of male-advantage genes, like those for male sex determination and fertility [4,5,12,13,14,15,16,17,18]. In addition, Y chromosome genes are critically involved in development [19], and may be implicated in hypertension, immune response, cancer, and ageing [20,21,22,23]. Since the Y chromosome is non-recombining and is clonally inherited through the male lineage, MSY polymorphisms are used to trace patrilines and the evolutionary history of populations [24,25,26,27,28,29].

Lack of recombination also increases the rate that mutations accumulate, making Y the fastest evolving chromosome in the genome [4,5,30,31]. The evolutionary dynamics of the Y chromosome involves acquisition, loss, and amplification of genes and genomic regions, resulting in structural complexity and broad variation across mammalian species [4,10,14,16,18,32,33]. These features complicate MSY sequence assembly and to date, only three primate [16,33,34] and the mouse [17] MSYs are complete, with high-quality MSY draft assemblies available for another handful of mammalian species (gorilla [35], marmoset [19], rat [19], dog [32], cat [32], pig [16], bull [19,36,37], horse [18], and opossum [19]), and partial MSY sequences have been obtained for a few more domestic and wild species (goat [38], sheep [27,29], red fox [39], brown and polar bears [40,41], and whales [42]).

Camelids, including the alpaca (*Vicugna pacos*), represent a mammalian group of particular biological, biomedical, evolutionary, economic, and cultural interest. All camelid species are adapted to extreme environments [43,44], present unique and unusual features of the adaptive immune system [45], and form a basal clade in the Cetartiodactyla phylogenetic tree [46]. They are also important production species and icons of cultural legacy in their regions of origin [47,48]. These interests are reflected by recently elevated activities in camelid genomics resulting in chromosome-level reference genomes for the alpaca [44] and dromedary [49], though providing no information about the Y chromosome because both genomes were derived from female individuals. Thus far, Y chromosome studies in South American camelids are limited to population analyses using partial sequences of *SRY*, *ZFY,* and *DBY* (*DDX3Y*) [50]. The Bactrian camel Y chromosome has received more attention since male individuals were used for two reference assemblies [51,52] and more recently, MSY sequence variants were identified from select PCR amplicons [53] and from a 3.8 Mb partial MSY assembly [28] for population studies. Despite these efforts, there is no annotated MSY reference assembly for any camelid species, leaving their reference genomes incomplete and missing a biologically, evolutionarily and economically important part of the genome.

The present study aims to start filling this gap in the alpaca reference genome. We present an 8.22 Mb assembly of the alpaca Y chromosome, derived from long- and short-read sequence data of flow-sorted Y chromosomes of a single male. The assembly includes 4.48 Mb of PCR-validated MSY and 3.74 Mb of the pseudoautosomal region in Y. The assembly is characterized for sequence features and annotated for genes using testis RNAseq and sequence data of the Y-enriched testis transcriptome obtained by hybridization capture and cDNA selection. The cytogenetic and molecular features of the alpaca Y chromosome are compared with those of other camelids and mammals.

## 2. Materials and Methods

### 2.1. Ethics Statement

Procurement of blood and skin biopsies for cell cultures and testis tissue for RNAseq followed the United States Government Principles for the Utilization and Care of Vertebrate Animals Used in Testing, Research and Training. These protocols were approved as AUP #2018-0342 CA (approved October 7, 2020) at Texas A&M University, TX, USA.

### 2.2. Samples and DNA Isolation

Peripheral blood samples in EDTA- and Na-heparin vacutainers (BD Vacutainer^®^) and/or skin biopsies were obtained from multiple male and female alpacas, dromedaries (*Camelus dromedarius*) and Bactrian camels (*Camelus bactrianus*) in the course of regular cytogenetic testing at Texas A&M Molecular Cytogenetics lab. In addition, testis tissues were obtained from two male alpacas during scheduled castration.

Genomic DNA (gDNA) of male and female camelids was isolated from EDTA-stabilized peripheral blood using Gentra Puregene Blood Kit (Qiagen, Hilden, Germany) following the manufacturer’s protocol. The DNA was checked for quality and quantity with Nanodrop 2000 spectrophotometer (Thermo Scientific, Waltham, MA, USA).

### 2.3. Cell Cultures and Cytogenetics

Primary fibroblast cell lines were established from skin biopsies from a male Huacaya alpaca and a male dromedary camel. The cells were grown in standard conditions at 37 °C with 4% CO_2_ in a culture medium containing α-MEM with Glutamax and nucleosides (Gibco, Dublin, Ireland), 20% fetal bovine serum (FBS; Atlanta Biologicals, Minneapolis, MN, USA), and 1× antibiotic-antimycotic (100 ×; Invitrogen, Carlsbad, CA, USA). Most of the cultures were grown to confluency and frozen in liquid nitrogen (LN2) in batches of 1 million cells per 1 mL freezing media (α-MEM, 10% DMSO, 10% FBS, 1× antibiotic-antimycotic) for future needs. Actively proliferating fibroblast cultures from the two species were also harvested for chromosome preparations following standard protocols described elsewhere [54]. Chromosome preparations for additional male alpacas, dromedaries and Bactrian camels were obtained from short-term peripheral blood lymphocyte cultures following standard procedures [54]. The preparations were used for cytogenetic analysis and stored at −20 °C for fluorescence in situ hybridization (FISH).

Primary fibroblast and/or blood lymphocyte cultures from all individuals were karyotyped by Giemsa staining and/or GTG-banding [55] with an Axioplan2 microscope (Carl Zeiss, Inc., Jena, Germany) and IKAROS (MetaSystems GmbH, Altlussheim, Germany) software, following the nomenclatures proposed for the alpaca [56] and the dromedary [57].

Identification of the Y chromosome and the smallest pair of autosomes (chr36) was confirmed by FISH with alpaca pseudoautosomal region (PAR) and chr36 specific BAC clones [56] following the standard protocol [54] (see also Section 2.4).

### 2.4. Flow-Sorting Alpaca and Dromedary Y Chromosomes, Y DNA Amplification and Validation by FISH

Flow-sorting of Y chromosomes from fibroblast cell lines of a male alpaca and a male dromedary was done on a dual-laser cell sorter (MoFlo, Beckman Coulter, Brea, CA, USA) at the Cambridge Resource Centre for Comparative Genomics (Cambridge, UK), following standard procedures [58]. Approximately 20,000 Y chromosomes were obtained for each species.

Flow sorted Y DNA was amplified with REPLI-g Single Cell kit (Qiagen, Hilden, Germany) according to the manufacturer’s protocol and the amplified product was cleaned using the QIAquick PCR purification kit (Qiagen, Hilden, Germany). Molecular weight of the products was evaluated on a 2% agarose gel and by pulsed field gel electrophoresis (PFGE). One microgram of the alpaca and dromedary Y DNA was labeled with biotin and/or digoxigenin by nick translation using Biotin or DIG Nick Translation Mix, respectively (Roche Diagnostics, Penzberg, Germany), and the manufacturer’s protocol.

In order to determine the efficiency of flow sorting and validate Y chromosome origin, labeled alpaca Y and dromedary Y were hybridized to alpaca, dromedary and Bactrian camel metaphase chromosomes following standard FISH procedures [54]. Hybridization signals of biotin-labeled probes were detected with avidin-FITC (Vector Laboratories, Burlingame, CA, USA) and digoxigenin-labeled probes with anti-digoxigenin Rhodamine (Roche Diagnostics, Penzberg, Germany). Chromosomes were counterstained with 4′,6-diamidino-2-phenylindole (DAPI). Images were captured and analyzed on a Zeiss Axioplan 2 fluorescence microscope using Isis V5.2 (MetaSystems GmbH, Altlussheim, Germany) software.

### 2.5. Sequencing and Assembly of the Flow-Sorted Alpaca Y Chromosome

Short-read sequencing of flow-sorted and amplified alpaca Y DNA was done on the Illumina MiSeq platform as paired-end 200 bp reads. The quality of sequence reads was analyzed with Trim Galore! (https://github.com/FelixKrueger/TrimGalore): the last 15 bp of every read was removed, followed by trimming with Q30 as a quality score cut off. For long-reads, the same alpaca Y DNA was sequenced on a PacBio Sequel 2 platform. Quality check and assembly of long PacBio raw reads was done with CANU [59] and Illumina MiSeq reads were incorporated into the PacBio assembly with PILON for further error correction [60]. The genomic assembly was analyzed for GC content and repeats with RepeatMasker (http://www.repeatmasker.org/).

### 2.6. Testis RNA Isolation, cDNA Library Preparation and Hybridization Capture of Y-Specific Transcripts

Total RNA was extracted from RNAlater (Invitrogen, Calrsbad, CA, USA) preserved testis tissue using RNeasy Mini Kit (Qiagen, Hilden, Germany) and mRNA was separated from total RNA with NEXTflex Poly(A) beads (BIOO, Austin, TX, USA). Illumina compatible cDNA libraries were generated with NEXTflex Rapid Directional qRNA-Seq kit (BIOO, Austin, TX, USA). All three steps were conducted following the manufacturers’ protocols. The libraries were cleaned with AMPure XP beads (Agencourt, Beverly, MA, USA) and checked for quality on Tape Station (Agilent, Santa Clara, CA, USA) using a 1 kb high sensitivity tape.

The libraries were hybridized to biotin labeled alpaca Y (see 2.4) as follows. One µg of the cDNA library was combined with 2 µg of alpaca Cot1 DNA in a volume of 10 µL with DNase/RNase free water and mixed with an equal volume of 2 × hybridization mix consisting of 10 mM EDTA, 0.2% SDS, 40 mM NaH_2_PO_4_/ Na_2_HPO_4_ (pH 7.2), 10× Denhardt’s solution, and 1.5 mM NaCl. The reaction was denatured for 5 min at 100 °C and allowed to pre-hybridize for 4 h at 65 °C to block repeats. Next, 100 ng of biotin labeled alpaca Y was combined with 5 µL of 2× hybridization mix, the volume was brought to 10 µL with H_2_O and over-laid with 50 µL of mineral oil. In a thermocycler, Y hybridization mix was denatured for 5 min at 100 °C, brought to 65 °C, combined with the prehybridized cDNA libraries, and allowed to hybridize for 72 h at 65 °C. After hybridization, the biotin-Y-cDNA mixture was combined with 100 µL of Dynabeads™ M-280 Streptavidin (Thermo Fisher, Waltham, MA, USA), the beads were washed and hybridization products were eluted as described previously [61]. The elution products were re-amplified and cleaned one more time with AMPure XP beads (Agencourt, Beverly, MA, USA), and the captured Y-enriched cDNA libraries were checked for quality on Tape Station 1 kb high sensitivity tape.

### 2.7. Sequencing and de Novo Assembly of Y-Enriched Cdna Libraries

The Y-enriched testis cDNA libraries were sequenced on an Illumina MiSeq platform, with paired-end 200 bp reads. The last 10 bp were trimmed off every read and sequences that did not meet Q30 quality cut-off were removed using TrimGalore! Illumina cDNA reads were assembled with Trinity [62] using default parameters.

### 2.8. Y-Enriched Testis Transcriptome Annotation

Transcripts were annotated with Trinotate [63], open reading frames (ORFs) were predicted with Transdecoder [62] and homology with known mammalian Y sequences was determined by BLASTp and BLASTx (https://blast.ncbi.nlm.nih.gov/Blast.cgi) with a cutoff for significant similarities as an E-value equal or smaller than 1 × 10^–5^ for initial identification. Additional functional information about the transcripts was searched through the Pfam database (https://pfam.xfam.org/) and by using TmHMM [64], SignalP [65], RNAmmer [66], and smartBLAST (https://blast.ncbi.nlm.nih.gov/smartblast/) tools. Also, alpaca Y transcripts were manually searched for homology to known eutherian MSY and PAR genes using the horse MSY gene catalogue [13,18] as well as the gene catalogue of the dromedary X chromosome (NCBI Genome: https://www.ncbi.nlm.nih.gov/genome/) to drive discovery.

All assembled transcripts were analyzed by BLAST against a locally downloaded non-redundant nucleotide database from GenBank with BLASTplus with a cutoff for significant similarities as an E-value smaller than 1 × 10^–25^. Transcripts that were not recognized were re-analyzed by discontiguous MegaBLAST against GenBank to reveal less similar sequences using the same criteria for significance. Transcripts with no significant similarity were considered as putative novel Y-specific, were sorted by size and masked for repeats with RepeatMasker (http://www.repeatmasker.org/). All putative Y transcripts with BLAST results, as well as all transcripts with no significant similarity, were further analyzed by BLAST against the alpaca Y genomic assembly to discern gene models and determine whether these sequences were single copy or multi-copy. The transcripts that were not found in the alpaca Y assembly were removed from further analysis. Gene models were further refined by aligning confirmed MSY transcripts as well as previously generated alpaca testis RNAseq data [44] to alpaca Y sequences using default parameters with HISAT2 [67]. Results were output as BAM files (.bam), which were then sorted and indexed with SAMtools [68] and loaded into IGV genome browser in order to visualize the gene models and coverage.

Amelogenin Y (*AMELY*) was located by discontiguous MegaBLAST analysis of human (NM_001143.2), cattle (NM_174240.2), pig (NM_213800.1), and goat (NW_017189585.1) *AMELY* transcript sequences against alpaca Y assembly.

### 2.9. Validation of the Alpaca Y Assembly and Putative Y Transcripts

Putative MSY and PAR contigs were identified by discontiguous megaBLAST analysis of the assembly against VicPac3.1 [44] with significant similarity defined as greater than or equal to 95% identity over at least 25% of the length of the contig. Contigs without significant similarity were considered putatively MSY, whereas contigs with significant homology to the alpaca X chromosome were considered putatively PAR. Contigs with significant similarity to VicPac3.1 scaffolds other than the X chromosome were considered autosomal contamination during flow-sorting and were removed from analysis. Contigs shorter than the mean read length of the PacBio subreads of the flow sorted Y were also removed from analysis.

The alpaca Y genomic assembly (putative Y contigs and all contigs over 200 kb) and the transcripts that were located in the alpaca Y assembly were validated by PCR. Primers (Table S1) were designed with Primer Quest (Integrated DNA Technologies, Coralville, IA, USA). The primers were first checked against female reference VicPac2 by in silico PCR in the UCSC Genome Browser (https://genome.ucsc.edu/) to remove autosomal contaminants, and against the gene models to remove those spanning splice sites. Finally, primers were located in the alpaca Y assembly using the ‘blastn-short’ option of BLASTplus in order to determine continuity with earlier BLAST results of the transcripts. The final primers were tested by PCR using 5× FIREPol^®^ Ready to Load Master Mix (Solis BioDyne, Tartu, Estonia) on gDNA of two male and two female alpacas as the templates. The PCR products were resolved in 2% agarose gel. Differential amplification between males and females, and amplification of a product of expected size were considered as indications of Y-specificity and correct assembly, respectively. For comparative purposes, all male-specific primers were tested by PCR on male and female gDNA of dromedaries and Bactrian camels.

### 2.10. Quantitative Pcr (Qpcr) Analysis of Putative Multi-Copy Sequences

Alpaca Y genes that showed more than one full copy when compared to the alpaca Y assembly, were considered potentially multi-copy and subject for analysis by qPCR. Primers for qPCR were designed with Primer Quest tool (Integrated DNA Technologies, Coralville, IA, USA) and checked for male specificity by in silico PCR in female assembly VicPac2 (UCSC: https://genome.ucsc.edu/) with the result “no significant similarity” suggesting Y-origin. The *UTY* gene was chosen as a single copy control. Primers were optimized by standard PCR on gDNA of two male alpacas as described in Section 2.9. The qPCR experiments were performed with LightCycler 480 (Roche Diagnostics, Pennzberg, Germany) in duplicate assays. Each assay was done in triplicate 20 µL reactions containing 50 ng of template DNA, 10 mM primers and 5x Hot FIREPol^®^ EvaGreen^®^ qPCR Mix Plus (Solis Biodyne, Tartu, Estonia). The cycling conditions were one cycle 5 min at 95 °C; 45 cycles 10 s at 95 °C, 5 s at 58 °C, and 10 s at 72 °C; one cycle for melting curve 30 s 95 °C, 30 s 65 °C, and final cooling for 20 s at 50 °C. Relative copy numbers were determined in comparison to the single-copy reference gene (*UTY*). Copy numbers were quantitated by the 2ΔΔCt method [15].

### 2.11. Construction of Neighbor-Joining Trees for Alpaca Y Genes

Neighbor-joining distance trees were obtained for all confirmed alpaca Y genes as follows. Transcript sequences were analyzed by megaBLAST, 50 closest related GenBank sequences were downloaded locally as fasta files, duplicate results from the same species were removed, best hits to 20 species were imported into MEGA10 (https://www.megasoftware.net/), converted to MEGA format, and neighbor-joining trees were built using standard parameters. Trees were mid-rooted with camelids as the outgroup.

## 3. Results

### 3.1. Y Chromosome Cytogenetics and Flow-Sorting

**Figure 1:**
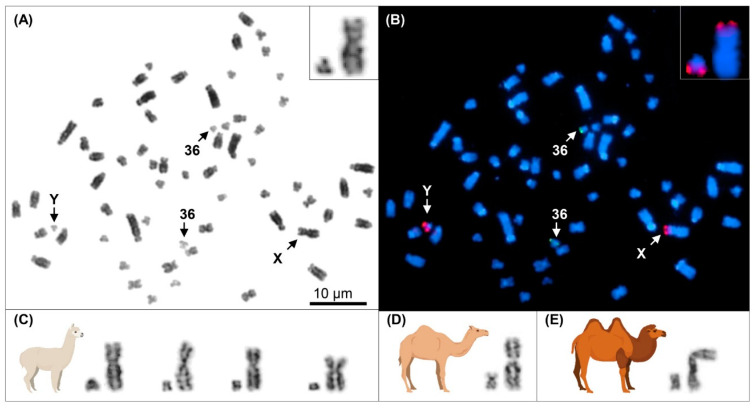
Cytogenetics of camelid sex chromosomes. (**A**) Giemsa stained alpaca metaphase spread showing (arrows) table 36. (**B**) The same metaphase after FISH with PAR BAC 21N1 (red) and chr36 BAC 3N14 (green) (Avila et al. 2014); Y and X with PAR signals are enlarged at upper right; (**C**) Alpaca Y and X chromosomes from 4 different cells; (**D**) Dromedary Y and X chromosomes, and (**E**) Bactrian camel Y and X chromosomes.

Cytogenetic analysis of metaphase spreads of male alpacas showed that the alpaca Y chromosome was the smallest element in the alpaca karyotype, similar in size or slightly smaller than the smallest autosome, chr36, and about 15% of the X chromosome (Figure 1A–C). Given that the current draft assemblies of most domestic animal X chromosomes range between 120 Mb and 150 Mb (https://www.ncbi.nlm.nih.gov/genome/), we predicted the approximate molecular size of alpaca Y as 15–20 Mb. Due to the small size and absence of distinct cytogenetic features, the morphology and centromere position of alpaca Y remained uncertain. Inspection of the Y chromosome in multiple cells by Giemsa staining and consecutive FISH with PAR BACs (Figure 1A,B) suggested that alpaca Y is submetacentric with a very small short arm (p-arm) and the PAR located terminal in the long arm (q-arm; Figure 1B). Comparison of alpaca Y with those of Old-World camels showed that the alpaca Y chromosome is the smallest, approximately half the size of the dromedary Y and 1/3 of the Bactrian camel Y chromosome (Figure 1C,D).

We isolated by flow sorting 20,000 alpaca and 20,000 dromedary Y chromosomes. Following amplification, this resulted in high molecular weight alpaca and dromedary Y DNA with the majority of fragments in the range of 10 kb to 35 kb (Figure S1). This material was suitable for labeling, cDNA selection, FISH, and both long-read and short-read sequencing. The fragment size distribution was; however, not adequate for the construction of fosmid or mini BAC libraries for the Y chromosomes of either species.

**Figure 2:**
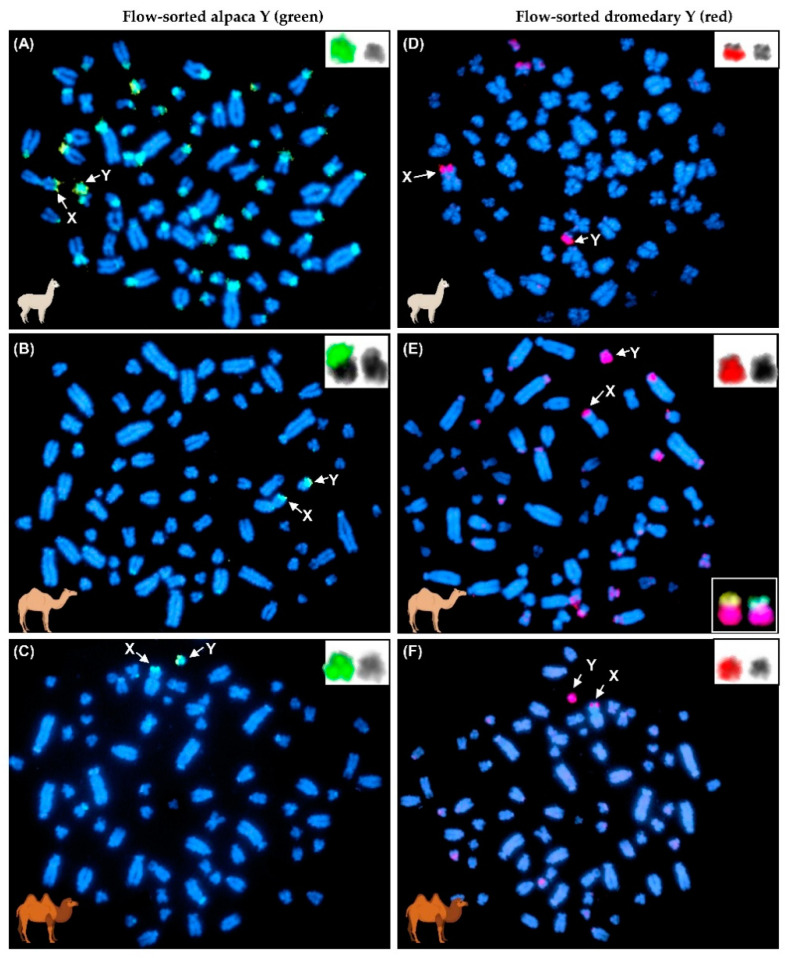
Validation of flow-sorted alpaca Y (A–C) and dromedary Y (D–F) by FISH. Partial metaphase spreads of male alpaca (**A**,**D**), dromedary (**B**,**E**) and Bactrian camel (**C**,**F**); the Y and the X chromosomes are denoted by arrows; windows at the upper right corner of each metaphase show enlarged Y chromosomes, one merged with FISH painting signal, another as inverted DAPI image. In (**E**), a window in the lower right corner shows dromedary Y chromosomes from two different cells co-hybridized with alpaca Y (green) and dromedary Y (red).

Before any further applications, the origin and specificity of flow-sorted and amplified alpaca Y was validated by FISH on alpaca, dromedary and Bactrian camel metaphase spreads (Figure 2A–C). For comparative purposes, similar FISH experiments were conducted with the flow-sorted dromedary Y (Figure 2D–F). The alpaca Y probe specifically painted the alpaca Y chromosome and the X-PAR, but produced additional and consistent FISH signals in the majority of autosomal centromeres and in heterochromatic short arms of many autosomes (Figure 2A). In contrast, alpaca Y specifically hybridized to the short arm of dromedary Y, both arms of Bactrian camel Y and the X-PAR in Old-World camelids, with only a few weak signals in select autosomal centromeres (Figure 2B,C). The flow-sorted dromedary Y probe was specific to the entire dromedary and Bactrian camel Y, painted the long arm of alpaca Y, gave signal in the X-PAR in all three species, and produced a few additional signals at some autosomal centromeres (Figure 2D–F). In summary, the FISH results confirmed that flow-sorting of alpaca and dromedary Y chromosomes was successful and that the material was predominantly specific to the Y chromosome and the PAR. The results also showed that alpaca Y shares repetitive sequences with alpaca autosomes but not with dromedary or Bactrian camel autosomes, and that the repeat content of alpaca and dromedary Y chromosomes is different. Finally, the FISH results agreed and refined our cytogenetic observations about camelid Y chromosomes (Figure 1) showing that alpaca Y is the smallest and homologous only to the short arm of dromedary Y (Figure 2E). Importantly, successful FISH with flow-sorted alpaca and dromedary Y chromosomes in all three camelid species indicated high degree of evolutionary conservation of the camelid Y chromosome.

### 3.2. Alpaca Y Genomic Assembly and Analysis

The genomic sequence of flow-sorted alpaca Y was assembled in two steps. First, we assembled 770,343 long, but error-prone PacBio reads (mean length 8034 bp; N50 6392 bp) and next, combined this with 11,883,537 short, but more accurate Illumina reads to improve assembly correctness. The resulting 20 Mb assembly was comprised of 652 contigs with a size range from ~1000 bp to ~1.4 Mb (Table 1, Figure S2). From this initial assembly, we removed approximately 1.8 Mb comprised of contigs that were shorter than mean read length of PacBio data. Another 1,420,848 bp (93 contigs) were removed due to homology to autosomal or unplaced scaffolds in VicPac3.1. Of the remaining assembly, 5,598,446 bp (33 contigs) showed significant (>95% identity of >25% length) homology to alpaca X and 11,190,354 bp (99 contigs) were putatively considered to be of Y-origin because they showed no significant similarity to the female assembly VicPac3.1.

Analysis by PCR of the 30 largest putative Y contigs (cutoff size 200 kb) and all contigs containing putative Y genes (Table S1; Figure S3) confirmed 18 contigs with a cumulative size of 4.48 Mb corresponding to male-specific Y (MSY) (Table 2). Of these, tig419 (~300 kb) was only partially male-specific suggesting that this contig spans the pseudoautosomal boundary in Y (PAB-Y). In addition, 13 contigs with a cumulative size of 3.74 Mb (Table S2) where identified as PAR due to significant homology with alpaca X scaffolds ScfyRBE_77259; HRSCAF = 79152 and ScfyRBE_77260; HRSCAF = 79153 in VicPac3.1 [44], and these contigs contained genes that have been assigned to the alpaca PAR by FISH [56]. Altogether, we confirmed Y origin of 31 contigs with a cumulative size of 8.22 Mb. The 18 MSY contigs were subject to detailed annotation with transcriptome assembly, while the remaining contigs showing PCR amplification in males and females were not further analyzed in this study.

### 3.3. Transcriptome Assembly and Msy Annotation

The assembly of short reads from Y-enriched testis cDNA libraries produced 154,194 transcripts with a size range from 201 bp to 11,199 bp, and an average length of 504 bp (Table S3; Figure S4). Approximately 24% of assembled transcripts, had ORFs longer than 99 amino acids (aa), and the maximum and average ORF sizes in this group were 2560 aa and 166 aa, respectively.

**Figure 3:**
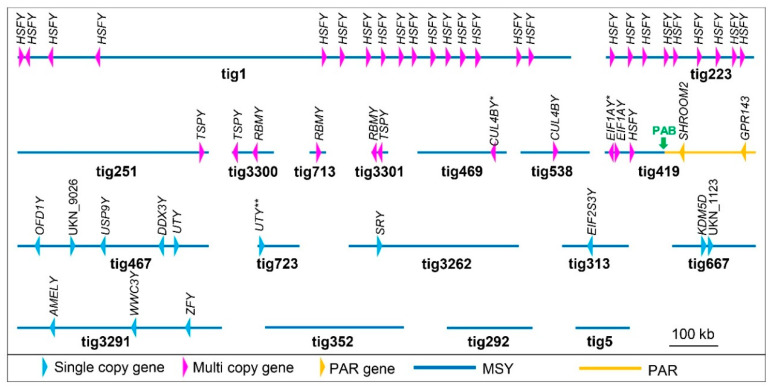
Alpaca Y contigs and mapped genes. Schematic drawings of 18 MSY contigs showing the relative position and orientation (arrowheads) of annotated genes and transcripts. The length of contigs and the position of genes are drawn to the scale; PAB—pseudoautosomal boundary; *—truncated copies of *EIF1AY* and *CUL4BY*; **—single copy *UTY* is split between two contigs.

Analysis of assembled transcripts by BLASTp produced 6785 unique hits with significant similarity (<1e−5), including hits to 25 known mammalian MSY and 21 putative camelid PAR genes (Table S4), while 3699 transcripts did not show significant similarity to any known GenBank sequences. In order to identify strictly male-specific transcripts, all transcripts with significant BLASTp hits and 220 largest transcripts (753 bp–3210 bp) with no significant similarity, were analyzed by discontiguous MegaBLAST against the 4.48 Mb alpaca Y assembly (Table 2). Altogether, we identified 15 known eutherian MSY genes and 2 novel transcripts (Table 3) which mapped to 14 of the 18 male-specific Y contigs (Figure 3).

**Figure 4:**
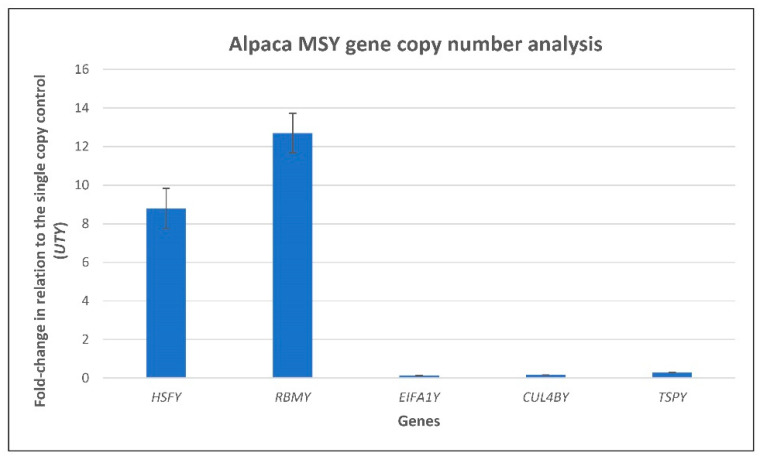
Copy number analysis of MSY genes. Relative quantitation of *HSFY*, *RBMY*, *EIF1AY*, *CUL4BY*, and *TSPY* (x-axis) copy numbers in relation to *UTY* as a single copy control (fold change; y-axis); vertical black bars at the top of fold-change columns denote standard deviation.

Alignment of assembled transcripts with MSY contigs, allowed the determination of copy numbers and gene models for MSY genes (Table 3). Five genes, *TSPY*, *RBMY*, *HSFY*, *EIF1AY*, and *CUL4BY*, had two or more copies (Figure 3). The highest copy number (26) was observed for *HSFY* which was present as 15 directional and one inverted copy in tig1, nine directional copies in tig223, and a single copy in tig419 (Figure 3). Relative copy numbers of *HSFY*, *RBMY*, *EIFA1Y*, *CUL4BY* and *TSPY* genes were validated by qPCR showing over 12-fold increase for *RBMY* and over eight-fold increase for *HSFY* compared to a single copy control gene, *UTY* (Figure 4). However, *TSPY* showed just a marginal fold increase and no copy number differences were observed for *EIF1AY* and *CUL4BY* compared to *UTY* (Figure 4). For the remaining genes and novel transcripts, we identified only a single copy in the PCR-validated MSY assembly, whereas the single copy *UTY* was split between tig467 (exons 1–16) and tig723 (exons 17–28) (Figure 3). Notably, alpaca Y has retained linkage of *USP9Y*-*DDX3Y*-*UTY* (see tig467, Figure 3), known to be conserved across eutherian MSY [10,18,19,32].

Gene models were determined for all single copy genes and individual copies of multi copy genes (Table 3), except for *AMELY*. This was because *AMELY* has very low or no expression in testis [16,37] and could not be annotated with alpaca testis transcripts. Instead, it was annotated using human, cattle, pig, and goat *AMELY* transcripts. The resulting alignments allowed to recognize the location of the gene in tig3291 but were not sufficient for determining its exon-intron structure (Table 3). Among multi copy genes, individual copies of *HSFY*, *TSPY*, and *RBMY* had the same exon-intron structure and were considered complete. However, we observed additional short (<200 bp) sequences with homology to *RBMY*, likely representing incompletely assembled copies or pseudogenes. Furthermore, *EIF1AY* had one full copy with seven exons and another, truncated and inverted copy with exons 1–4, both in tig419 (Figure 3), suggesting the presence of a truncated pseudogene or error in the assembly. Similarly, *CUL4BY* appeared as one full copy with 8 exons in tig538 and as short sequences homologous to *CULB4Y* in tig469. The gene models were further validated by PCR with primers designed from predicted exons (Table S1) and in most cases male-specific PCR products of expected size were observed (Figure S3). The exception was *DDX3Y*, where primers amplified male-specific but a larger product than expected, suggesting incorrect assembly or an error in determining exon-intron structure.

### 3.4. Demarcation of PAR-Y and Putative Pseudoautosomal Boundary (PAB)

**Figure 5:**
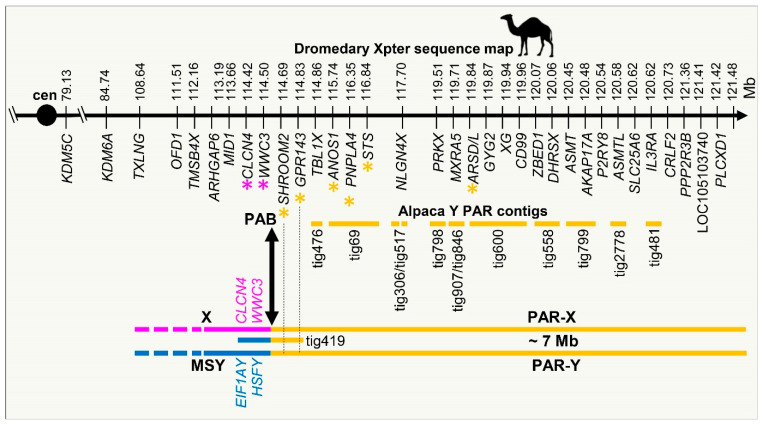
Reconstruction of the PAR in both sex chromosomes. The sequence map of the terminal region of dromedary Xp (Xpter) is presented at the top; orange horizontal lines denote PAR-X, PAR-Y, and alpaca Y PAR contigs; the location of PAB proximal to *SHROOM2* is denoted with a black arrow; purple lines and fonts denote X-specific sequences and genes, and blue lines and fonts Y-specific sequences and genes; gene symbols with a star (*) denote the genes that have been mapped to alpaca and dromedary PAR by FISH [56].

Annotation of the alpaca Y assembly (see Section 3.2) assigned 13 contigs (3.74 Mb) to PAR (Table S2), of which tig419 spanned the pseudoautosomal boundary in Y (PAB-Y) (Figure 3 and Figure 5), so that approximately half of this contig (1–177,307 bp) shared 99.1% sequence similarity with the alpaca X chromosome in VicPac3.1 [44], while no similarity was found for the remainder. The current alpaca X chromosome assembly is highly fragmented and incomplete [44], so we combined information on the gene content of the 13 PAR-Y contigs (Table S2) with a sequence map of Xp terminal region (Xpter) in the recent dromedary reference CamDro3 [49], and reconstructed the likely span and gene content of the alpaca PAR on both sex chromosomes (Figure 5). Sequence alignment of *SHROOM2* from the alpaca Y (this study) and X chromosomes (VicPac3.1) showed 98.4% similarity suggesting that this is the last PAR gene before the sex chromosome specific regions start. We tentatively located the PAB between *SHROOM2* and *WWC3* in the X chromosome, and between *SHROOM2* and *HSFY* in the Y chromosome. Even though a previous study mapped a BAC clone containing *WWC3* to alpaca and dromedary PAR [56], our results did not support this as we did not find *WWC3* sequences in tig419. Instead, the gene was found in a Y-specific contig tig3291 together with *ZFY* and *AMELY* (Figure 3) and after confirming male-specificity by PCR (Figure S3), was denoted as *WWC3Y*. Based on the demarcated PAB and the dromedary X chromosome sequence map [49], we estimated the approximate size of the alpaca/camelid PAR to be 7 Mb. Thus, the 3.74 Mb of PAR contigs represented 54% of the region in the Y chromosome.

### 3.5. Comparative Analysis of Alpaca Y Transcripts

**Figure 6:**
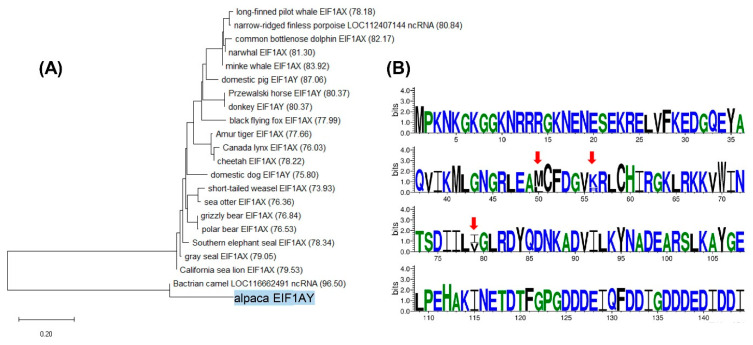
Comparative analysis of alpaca *EIF1AY*. (**A**) Neighbor-joining nucleotide tree showing the closest related sequences to the alpaca *EIF1AY* in Genbank as revealed by BLAST. Branch length is equivalent the relative distance between sequences. (**B**) Logographical representation of amino acid sequence conservation between human, mouse, and zebrafish orthologs of alpaca *EIF1AY* as revealed by smartBLAST. Residues at each position are scaled according to their frequency (y-axis; bits). The x-axis shows amino acids and their sequence positions. Residues are colored according to their biochemical properties: hydrophilic in blue, neutral in green, and hydrophobic in black. Positions with a single letter denote conservation across all species. Variable amino acids are indicated by red arrows and the size of the two letters in these positions is proportional to their occurrence across all species.

Analysis of alpaca MSY gene transcripts against GenBank by discontiguous megaBLAST, revealed the most similar mammalian sequences and allowed the construction of neighbor-joining trees for the 14 alpaca MSY X-degenerate genes (Table 3, Figure 6A, Figure S5). Most alpaca MSY genes clustered together with the corresponding orthologs of the Bactrian camel and the two species shared >90% homology with high sequence coverage (Table S5). This was expected because Bactrian camel is the only camelid species with publicly available male whole genome and assembled Y sequences [28,53,69,70]. However, because Bactrian camel Y is not annotated, several significant BLAST hits (<1e−25) with alpaca Y transcripts, were denoted as Bactrian camel X gametologs in Genbank. For example, BLAST analysis of alpaca *ZFY* transcript showed 97.28% of sequence identity with Bactrian camel *ZFX* (XM_032475351) (Table S5), but only 95.42% identity with alpaca *ZFX* (XM_015246765.2), indicating that the sequence denoted as Bactrian camel *ZFX* is actually *ZFY*. We confirmed this by PCR with primers designed from the Bactrian camel *ZFX* sequence (Table S1) and showed male-specific amplification in the Bactrian camel, as well as in the alpaca and dromedary (Figure S3).

In addition, we analyzed amino acid sequences of alpaca Y transcript ORFs by smartBLAST and identified the most similar amino acid sequences in human and mouse, and in zebrafish as an outgroup (Table S6). Similarity was the lowest for *RBMY* and *HSFY*, and the highest for two eukaryotic translation initiation factors—*EIF2S3Y* and *EIF1AY* (Table S6). An outstanding example of amino acid sequence conservation was *EIF1AY* with only three variable residues between the compared species (Figure 6B). Among these, position 50 was maintained in all but humans and position 56 was different in mice, but the same in other species. Position 79 was the same in alpaca and human and different in mouse and zebrafish (Figure 6B). Evolutionary conservation of *EIF1AY* ORF is even more notable because the gene is Y-linked in alpaca and human, but has been lost from the mouse Y chromosome [19] and is autosomal in zebrafish (NCBI Gene: https://www.ncbi.nlm.nih.gov/gene/).

## 4. Discussion

Here we present the first partial assembly of the alpaca Y chromosome, currently the only annotated MSY assembly in camelids. Flow sorted Y chromosomes were used and the material was free from apparent autosomal or X chromosome contamination as evidenced by FISH (Figure 2). Nevertheless, from the initially assembled ~20 Mb of putative Y chromosome (Table 1), only 40%, including 4.5 Mb of MSY (Figure 3) and 3.7 Mb of PAR-Y (Figure 5), were retained in the final assembly due to limited tools available to verify Y chromosome localization in camelids. For example, Y chromosome studies in other mammalian species clearly show that not all legitimate MSY sequences are strictly male specific by PCR and require validation by FISH [13,18]. Currently, the only BAC library for camelids is CHORI-246 (https://bacpacresources.org/), constructed from a female individual. Clones from this library can be used for assigning PAR sequences to the sex chromosomes [56], but not for MSY. The lack of a male-derived BAC library hinders the ability to verify MSY sequences by FISH, as well as, the use of a BAC tiling path for high-quality MSY sequence assembly, both of which have been possible in humans [16], pigs [71], horses [18], cattle [19], and cats and dogs [32]. FISH with male-specific BACs is essential for confirming the Y-origin of transposed sequences which have retained high degrees of sequence homology with their autosomal or X-linked counterparts, or to clearly discriminate between gametologs. We, therefore, excluded all sequences not verified by PCR as male-specific. The only exceptions were sequences that corresponded to known PAR genes based on alpaca or dromedary X chromosome assemblies [44,49] or prior FISH studies [56]. However, we anticipate that further improvement of the alpaca reference genome and generation of high-quality genome assemblies for multiple male individuals, will help to reveal additional true Y chromosome sequences among the 12 Mb of currently excluded contigs.

The MSY assembly presented here provides the first glimpse of the sequence content of the alpaca Y chromosome. A notable feature of alpaca Y was the extent of shared repetitive sequences with the autosomal genome (Figure 2A). The same was not observed between the alpaca, dromedary, and Bactrian camel Y chromosomes and the dromedary or the Bactrian camel autosomes (Figure 2B–F), suggesting the presence of unique repeat classes in the alpaca. A similar pattern has been described in equids where FISH with a horse Y chromosome-specific painting probe highlights repetitive sequences in multiple donkey autosomes, but not in the horse [72]. Shared repetitive content between the Y and autosomes in the alpaca was probably an additional factor complicating the validation of MSY sequences by PCR. Even though, all sequences used for primer design we masked for repeats, the RepeatMasker (http://www.repeatmasker.org/) database contains no alpaca or camelid-specific repeats. Thus, primers that amplified both in males and females could have been unknowingly designed in repeats, even though the sequences originated from MSY. Further evidence for repetitive content were ladder-like amplicons produced by some primers due to which the corresponding contigs were removed from further analysis. Other than the presence of these alpaca-specific repeats, the amount (48.3%) and content (predominantly L1 LINEs) of interspersed repeats in alpaca Y was comparable to those of other mammalian Y chromosomes, such as 45.7% in chimpanzee [33], 47% in gorilla [35], though slightly lower than the 54.3% in human [16,33], or 54% in horse [18]. Importantly, 1% of the alpaca MSY assembly is comprised of simple short tandem repeats or microsatellite sequences (Table 2), which if polymorphic, will be useful markers for the study of patrilines and population history in alpacas and other South American camelids. Likewise, the remaining 51.7% (2.3 Mb) of non-repetitive MSY will provide a reference for the discovery of Y-specific single nucleotide polymorphisms (SNPs), indels and copy number variants (CNVs) present in South American camelid populations.

It is well documented that the Y chromosome is the most dynamic and rapidly evolving part of the mammalian genome, with a tendency to lose ancestral genes, acquire and amplify genes from other chromosomes, and generate novel Y-born transcripts [14,16,18]. In this context, it was unexpected that in addition to 15 X-degenerate genes, we identified only two novel transcripts (Table 3) and no transposed genes. Though, we assume that due to the limitations to validate sequences of Y origin by PCR (see above), autosomal and X-transposed sequences and additional gametologs were likely removed from the analysis. For example, in the initial set of assembled transcripts, we found homology to 26 known mammalian MSY genes (Table S4). However, transcripts corresponding to 12 genes were not male-specific by PCR and were not included in the final gene catalogue. In contrast, the two novel transcripts UKN_1123 and UKN_9026 (Table 3), were clearly male-specific in alpaca and the two camels (Figure S3), though the nature of these sequences (transposons, noncoding RNA, or coding) or possible functions remain unknown. Even though both transcripts were derived from testis, and both had a single exon with a short ORF (Table 3), these features do not automatically attribute biological functions, mainly because the permissive epigenetic regulation of testis allows transcription of many potentially nonfunctional sequences [73]. A notable feature of the two novel transcripts, however, was that in contrast to the majority of novel Y-born transcripts described in humans [16], mice [17], carnivores [14,32], and equids [18], the alpaca novel transcripts were single-copy (Figure 3, Table 3), further questioning their potential functions.

Novel transcripts and acquired sequences from autosomes and the X chromosome are of particular interest regarding MSY evolutionary dynamics and functions in spermatogenesis and male fertility [10]. However, both types of sequences are typically specific to a species or a group of closely related species [14,16,17,18,33], and have limited value for comparative studies. The latter are predominantly based on the content and properties (i.e., single copy vs. ampliconic; functional vs. pseudogene) of X-degenerate genes or X-Y gametologs [3,18,19,32]. In alpaca MSY, we annotated 15 gametologs (Table 3, Figure 3, Table 4) out of the 39 currently known X-degenerate genes based on a recent comparison of 13 mammalian (12 eutherians and opossum) MSYs [18]. Of these, only *SRY* has been found in all species (Table 4) and our data suggest that, like in most species, alpaca *SRY* is a single copy gene (Table 4). Five alpaca MSY genes (*DDX3Y*, *TSPY*, *USP9Y*, *UTY*, and *ZFY*) are present in all 12 eutherians and four genes (*EIF1AY*, *EIF2S3Y*, *KDM5D*, and *RBMY*) in most species, with just a few exceptions. *AMELY* and *HSFY* are shared with eight, *OFD1Y* with five species, and *CUL4BY* has been found only in carnivores [32], pig [71], and equids [18] (Table 4). To date, *WWC3Y* has been unique to the horse MSY [18], while in other species the gene has remained X-specific. Therefore, it was a surprise to find male-specific *WWC3Y* in alpaca, mapping next to *ZFY* in tig3291 (Figure 3, Figure S3).

However, it is important to note that in this study we annotated alpaca Y using testis transcriptome only (Y-enriched testis cDNA libraries and alpaca testis RNAseq data), thus likely missing genes and transcripts with very low-level or no expression in testis. A good example is *AMELY*—a gene which is present in the Y chromosome of eight of the 12 eutherians (Table 4), but is predominantly expressed in tooth enamel [16]. Therefore, a special BLAST search was conducted for *AMELY* in this study. On the other hand, we probably did not miss much because the majority of eutherian Y genes and transcripts are expressed exclusively or among other tissues in testis [14,16,17,18,33,37,71]. The few known exceptions include human *TBL1Y* which is expressed in fetal brain and prostate, and *PCDH11Y* which is expressed in fetal and adult brain only [16], but the former is a PAR gene in camelids (Figure 5) and the latter is human-specific [16]. Nevertheless, once the alpaca reference genome is improved to high-quality and the Y assembly taken to scaffolds level, a more comprehensive annotation will be done using multi-tissue transcriptome data combined with in silico annotation with MAKER [74].

The present status of alpaca Y assembly with 18 contigs and 16 mapped genes/transcripts (Figure 3) provide no clues about the location or orientation of these sequences in the Y chromosome, and only limited information about the relative order of genes. The latter, as evidenced from high-quality MSY assemblies in primates [16,33,34,35,75], carnivores [32], rodents [17], pig [71], cattle [19,36], and horse [18], is not evolutionarily conserved and differs even between closely related species. The only MSY linkage group known to be conserved across all eutherians is that of *USP9Y*-*DDX3Y*-*UTY* [10,18,19,32]. Notably, conserved linkage of these three genes has also been retained in alpaca MSY (Figure 3). The reason for such evolutionary constraint, however, remains unknown.

One of the characteristic features of the Y chromosome is amplification of ancestral (X-degenerate) and acquired (transposed and novel) sequences, particularly those which have gained male benefit functions and need protection due to the lack of recombination in MSY [31,76]. Therefore, MSYs in many species are typically enriched with multicopy and ampliconic genes and transcripts (Table 4). However, our contig-level results indicate that the alpaca MSY is relatively depleted of such sequences. We were able to confirm multicopy status by sequence analysis and qPCR only for two genes—*RBMY* and *HSFY* (Figure 3, Figure 4, Table 4)—whereas sequence analysis showed more copies for *HSFY*, and qPCR more for *RBMY.* It is likely, that qPCR primers detected also the additional short sequences with homology to *RBMY* which were observed but not annotated in the assembled contigs. Sequence-based multicopy status of *TSPY*, *CUL4BY* and *EIF1AY* (Figure 3, Table 3) was, however, not confirmed by qPCR, though a tendency of increased copy number in relation to the single copy control was observed for *TSPY* (Figure 4). Since qPCR is limited to relative quantitation, accurate validation of alpaca MSY gene copy numbers will require digital droplet PCR analysis in future studies [77].

The discovery of at least 26 copies of *HSFY* in alpaca Y (Figure 3, Table 3) is of particular interest because this gene has been independently amplified in multiple species with the most extreme expansion observed in pig Y with at least 100 copies [78] and bull Y with at least 79 copies [37]. Multiple, though less copies of *HSFY* have also been found in human (two copies) and rhesus (three copies) [37], horse (three copies) [18], and cat (eight copies) [79]. It has been suggested that such recurrent independent amplification of *HSFY* during evolution is driving or carried along with a genomic conflict [78]. Indeed, recent super resolution sequencing of bull Y and part of the X chromosome shows that amplification of *HSFY* is accompanied by co-amplification of the X gametolog, *HSFX* (11 copies), and is a manifestation of sex-linked meiotic drive [37]. The phenomenon is known as X-Y arms races or X-Y coevolution through gene amplification, has been found in mouse and human, and is not limited to *HSFY/HSFX* [37]. To determine whether *HSFY* amplification in alpaca Y illustrates similar X-Y arms races, however, requires essential improvement of sex chromosome assemblies in this species.

While the primary focus of this study was MSY, it was expected that by sequencing the entire flow-sorted Y, we will also obtain PAR-Y sequences. Indeed, we identified 13 contigs containing 20 PAR genes (Table S2) and linearly ordered the contigs in the sex chromosomes using dromedary X chromosome sequence [49] as a template (Figure 5). The most notable finding, however, was the discovery of the pseudoautosomal boundary in Y (PAB-Y) in tig419 between a previously confirmed PAR gene *SHROOM2* [56,80] and Y-specific *HSFY* (Figure 3 and Figure 5). Furthermore, the discovery of alpaca male-specific *WWC3Y*, allowed the demarcation of PAB-X between *SHROOM2* and *WWC3* (Figure 5), thus refining earlier qPCR studies that suggested PAB-X to be between *SHROOM2* and *CLCN4* [80]. These findings provide valuable material for future studies to characterize alpaca PAB-Y and PAB-X sequence features in detail.

This study of alpaca MSY also expanded our comparative knowledge about the Y chromosome in camelids. All South American camelids (alpaca, llama, vicuna, guanaco) have a very similar, cytogenetically indistinguishable, diminutive Y chromosome [81] as represented by alpaca Y in this study, while the Y chromosomes of Old World camels are larger and morphologically distinct from that of the alpaca and from each other (Figure 1). Despite the cytogenetic differences and the estimated 11 to 25 million years of evolutionary distance between the New and Old World camelids (see [47]), this study demonstrated extensive sequence homology between the alpaca, dromedary and Bactrian camel Y chromosomes by FISH (Figure 2), by PCR (Figure S3), and by BLAST analysis of alpaca MSY genes (Figure 6 and Figure S5). Alpaca male-specific contigs, genes and transcripts, including the two novel transcripts, were male-specific also in camels. The only exception was tig292 (Figure 3), which did not amplify by PCR in the two camels (Figure S3), suggesting that the tiny alpaca Y has also some species-specific content. The extent of the latter, however, remains to be determined by future studies. Likewise, complete sequencing of the dromedary and Bactrian camel Y chromosomes is needed to reveal their extra content compared to alpaca Y and determine which of the Y chromosomes, the minimal Y of the alpaca or the largest Y of the dromedary, represents best the ancestral camelid Y chromosome.

## 5. Conclusions and Future Approaches

The 8.22 Mb iteration of the alpaca Y chromosome presented here is the first MSY assembly in New World camelids and the first annotated assembly of the Y chromosome in any camelid species. The contigs were produced by hybrid analysis of short and long reads from flow-sorted alpaca Y, demonstrating the accuracy of this approach for both assembly and cDNA capture. The utility for this data extends from population studies to genome evolution, as well as the potential discovery of factors affecting male fertility across camelids.

Though an essential step towards a complete genome, this contig-only version of alpaca Y is limited to contiguous portions of the MSY and PAR-Y. Their relative position to one another, distance between them, and content of the intervening and distal sequences are still obscured. Furthermore, due to the lack of genetic tools for camelids to confidently determine Y-origin of sequences, almost 12 Mb of putative Y contigs were not included in this reference, hindering a more complete result.

In that context, further work is needed to improve the assembly, even in the absence of aids such as a male BAC library. For a more complete and accurate assembly of the alpaca Y chromosome, the data generated in this study should be combined with high quality genome-wide hybrid assemblies (Illumina, PacBio, Oxford Nanopore) of a number of individual male alpacas. In addition, scaffolding techniques such as chromatin conformation capture using Hi-C or optical mapping using Bionano, can obviate the need for BAC libraries to help aid the assembly of complete chromosomes. For example, the recently developed techniques from the Telomere-to-Telomere (T2T) consortium [82] have produced chromosome-arm and whole-chromosome assemblies using a combination of long read technologies and the above scaffolding approaches. Lastly, the addition of long- and short-read data for more male individuals will aid our understanding of Y chromosome evolution and variation in camelids.

## Figures and Tables

**Table 1:** Summary metrics of assembled flow-sorted alpaca Y material from the combined PacBio and Illumina data.

Total Assembly	20,060,146 bp
Contig N50	288,719 bp
Contig L50	21
Number of Contigs	652
Largest Contig	1,433,950 bp
Smallest Contig	1142 bp
Mean Contig Length	30,767 bp

**Table 2:** Sequence features of alpaca MSY contigs (ordered by size); * PAB-Y: contains MSY and PAR.

Contig ID	Size, bp	GC, %	LINE, %	SINE, %	Simple Repeats, %	LTR, %	Total Repetitive, %
Tig1	1,120,224	35.8	43.43	0.08	0.84	9.77	54.2
Tig467	400,437	35.6	26.79	0.69	0.81	4.73	37.1
Tig251	381,152	44.6	18.14	1.90	1.1	6.17	32.8
Tig3291	348,639	36.5	41.52	0.71	0.92	4.64	50.4
Tig3262	337,762	36.5	38.74	0.37	1.63	24.03	65.2
Tig419 *	307,557	43.6	21.81	0.68	0.87	3.31	29.5
Tig223	297,467	33.3	67.38	0.16	1.48	2.76	72.0
Tig352	290,908	37.9	36.0	1	0.8	10.3	49.1
Tig667	169,226	37.1	35.47	0.19	1.43	7.32	46.3
Tig292	139,608	40.0	15.1	2.30	0.7	4.0	23.4
Tig469	136,307	36.60	58.56	0.11	0.48	2.28	62.76
Tig313	132,018	34.5	44.44	0.38	0.77	5.13	53.3
Tig5	124,853	34.9	31.4	0	1.3	13.4	46.4
Tig538	107,714	34.87	51.3	0.42	1.06	3.07	57.08
Tig3300	65,384	43.2	19.77	0.49	0.94	3.98	25.4
Tig723	64,517	33.85	30.60	1.07	0.55	2.97	40.02
Tig713	32,281	40.1	12.39	0	0	6.27	18.9
Tig3301	29,614	43.5	20.13	0	0.73	7.27	29.3
Total/Average	4,485,668	37.4	37.2	0.6	1	7.9	48.3

**Table 3:** Gene catalogue of the 4.48 Mb alpaca Y assembly.

Gene/Transcript Symbol	MSY Contig	Gene Category	No of Exons	Copy Number	Transcript Size, bp	ORF, aa
*AMELY*	tig3291	X-degenerate	n/a	1	n/a	n/a
*CUL4BY*	tig469, tig538	X-degenerate	8	2	1123	303
*DDX3Y*	tig467	X-degenerate	16	1	4873	474
*EIF1AY*	tig419	X-degenerate	7	2	2795	145
*EIF2S3Y*	tig313	X-degenerate	15	1	4588	472
*HSFY*	tig1, tig223, tig419	X-degenerate	2	26	1082	356
*KDM5D*	tig667	X-degenerate	26	1	10,322	1219
*OFD1Y*	tig467	X-degenerate	7	1	2182	450
*RBMY*	tig713, tig3300, tig3301	X-degenerate	4	3	3831	116
*SRY*	tig3262	X-degenerate	1	1	723	240
*TSPY*	tig251, tig3300, tig3301	X-degenerate	7	3	4880	140
UKN_1123	tig667	novel	1	1	994	104
UKN_9026	tig467	novel	1	1	773	143
*USP9Y*	tig467	X-degenerate	42	1	9928	2,489
*UTY*	tig467, tig723	X-degenerate	28	1	6366	913
*WWC3Y*	tig3291	X-degenerate	21	1	2109	246
*ZFY*	tig3291	X-degenerate	5	1	9574	509

**Table 4:** Comparative status of 15 alpaca X-degenerate genes in 12 eutherian mammals and opossum.

Alpaca Gene	Presence in Other Mammals
*AMELY*	horse, pig, cattle, cat, human, chimp, gorilla, rhesus
*CUL4BY* ^MC?^	horse ^MC^, pig ^MC^, cat ^MC^, dog ^MC^
*DDX3Y*	horse, pig, cattle, cat, dog, human, chimp, gorilla, rhesus, marmoset, mouse, rat
*EIF1AY* ^MC?^	horse, pig, cattle, cat, dog, human, chimp, gorilla, rhesus, marmoset
*EIF2S3Y*	horse, pig, cattle, cat, dog, mouse, rat
*HSFY* ^MC^	horse ^MC^, pig ^MC^, cattle ^MC^, dog, cat ^MC^, human ^MC^, gorilla ^MC^, macaque
*KDM5D*	horse, pig, cat, dog, human, chimp, gorilla, rhesus, marmoset, mouse, rat
*OFD1Y*	horse, pig, cattle, dog human ^PS^, chimp ^PS^, gorilla, macaque ^PS^
*RBMY* ^MC^	horse ^MC^, pig ^MC^, cattle, dog, human ^MC^, chimp ^MC^, gorilla ^MC^, rhesus, marmoset, mouse ^MC^, rat ^MC^
*SRY*	horse, pig ^MC^, cattle, cat, dog ^MC^, human, chimp, gorilla, rhesus, marmoset, mouse, rat ^MC^, opossum
*TSPY* ^MC?^	horse ^MC^, pig ^MC^, cattle ^MC^, cat ^MC^, dog ^MC^, human ^MC^, chimp ^MC^, gorilla ^MC^, rhesus ^MC^, marmoset, mouse ^PS^, rat ^MC^
*USP9Y*	horse, pig, cattle, cat, dog, human, chimp, gorilla, rhesus, marmoset, mouse, rat
*UTY*	horse, pig, cattle, cat, dog, human, chimp, gorilla, rhesus, marmoset, mouse, rat
*WWC3Y*	horse
*ZFY*	horse, pig, cattle, cat, dog, human, chimp, gorilla, rhesus, marmoset, mouse ^MC^, rat

Data retrieved from [18]; **^PS^**—pseudogene; **^MC^**—multicopy; **^MC?^**—possible multicopy.

## Data Availability

Data available in a publicly accessible repository.

## Supplementary Materials

The following are available online at https://www.mdpi.com/2073-4425/12/1/105/s1, **Figure S1.** Quality and quantity evaluation of flow-sorted and REPLI-g (Qiagen) amplified alpaca and dromedary Y chromosomes. (**A**) Agarose gel electrophoresis. Loaded 1 µL from 50 µL of REPLI-g reaction. Alpaca and dromedary Y quantity was about 800 ng/ µL; (**B**) pulse field gel electrophoresis (PFGE) analysis of alpaca and dromedary Y. Note that the majority of amplified DNA fragments range between 10 kb and 33.5 kb; NEB—New England Biolabs; **Figure S2.** Length distribution of the 652 contigs assembled from long and short reads of the alpaca flow sorted Y chromosome. The x-axis shows the length in base pairs; the y-axis shows the number of reads of a certain length, and the z-axis shows the cumulative percentage of reads of a particular size or shorter denoted by the orange line; **Figure S3.** (**A**) Images of agarose gel electrophoresis showing PCR results with primers designed from 18 MSY contigs and/or 12 specific genes (see Table S1 for primer sequences) in male (M) and female (F) gDNA of the alpaca (left), dromedary (middle) and Bactrian camel (right). The majority of primers produced male-specific PCR products of the expected size in all three species, thus confirming their MSY origin, correctness of the assembly and evolutionary conservation. The exceptions are shown in red font: primers for *DDX3Y* amplified different size products in alpaca and the two camels, and neither of these corresponded to the expected size (Table S1), though the amplification was male-specific in all three species; Primers for contig Tig292 did not amplify in the dromedary and Bactrian camel. (**B**) PCR results with primers designed from Bactrian camel sequence XM_032475351.1 denoted as *ZFX*. The primers show male-specific amplification in all three camelid species and suggest that this sequence corresponds to *ZFY*. 100 bp: molecular ladder by New England Biolabs; **Figure S4.** Length distribution of assembled transcripts with BLASTp results. The x-axis shows the length in base pairs, the y-axis shows the number of reads of a certain length, and the z-axis shows the cumulative percentage of reads of a particular size or shorter, denoted by the orange line; **Figure S5.** Neighbor-joining trees showing the closest related sequences to alpaca X-degenerate genes in Genbank as determined by discontiguous megaBLAST. (**A**) *EF2S3AY*; (**B**) *RBMY*; (**C**) *KDM5D*; (**D**) *OFD1Y*; (**E**) *CUL4BY*; (**F**) *HSFY*; (**G**) *TSPY*; (**H**) *SRY*; (**I**) *ZFY*; (**J**) *DDX3Y*; (**K**) *USP9Y*; (**L**) *UTY*, and (**M**) *WWC3Y*. All trees are mid rooted with the branch corresponding to alpaca. Branch length is equivalent to relative distance between two sequences. **Table S1.** Summary information of primers used to validate MSY contigs and assembly by PCR; * *DDX3Y* was male-specific in all three species but the amplicon was not of expected size; **Table S2.** Summary features of contigs identified as PAR by BLAST analysis against female assembly VicPac3.1; **Table S3.** Transcriptome assembly metrics (assembled with Trinity Assembler); **Table S4.** A list of known mammalian MSY and PAR genes (Janecka et al. 2018; Avila et al. 2014; Richardson et al. 2019; dromedary Xpter assembly in CamDro3) and the number of alpaca Y transcripts identified for each by BLASTp and BLASTx analyses (the two are not mutually exclusive); **Table S5.** Top hits for MSY transcripts in GeneBank by megaBLAST. Note that for 10 of the 14 genes, the top hit is from the Bactrian camel; **Table S6.** smartBLAST analysis results of alpaca MSY.

## Data Access

All sequencing data is available from the SRA at NCBI under Bioproject PRJNA681610 and Biosample SAMN16963716 with accessions to short-read DNA sequences, RNAseq and PacBio sequences as SRR13176458, SRR13176456, and SRR13176457, respectively.
