# Supplementary material for: An 8.22 Mb Assembly and Annotation of the Alpaca (Vicugna pacos) Y Chromosome
Source: Genes (Basel). 2021 Jan 16;12(1):105. doi: 10.3390/genes12010105 (PMC7830431; doi:10.3390/genes12010105)
Supplement: Supplementary file 1 [file genes-12-00105-s001.zip › Supplementary Material_R1/Supplementary Figures S1-S5_R1.pdf]

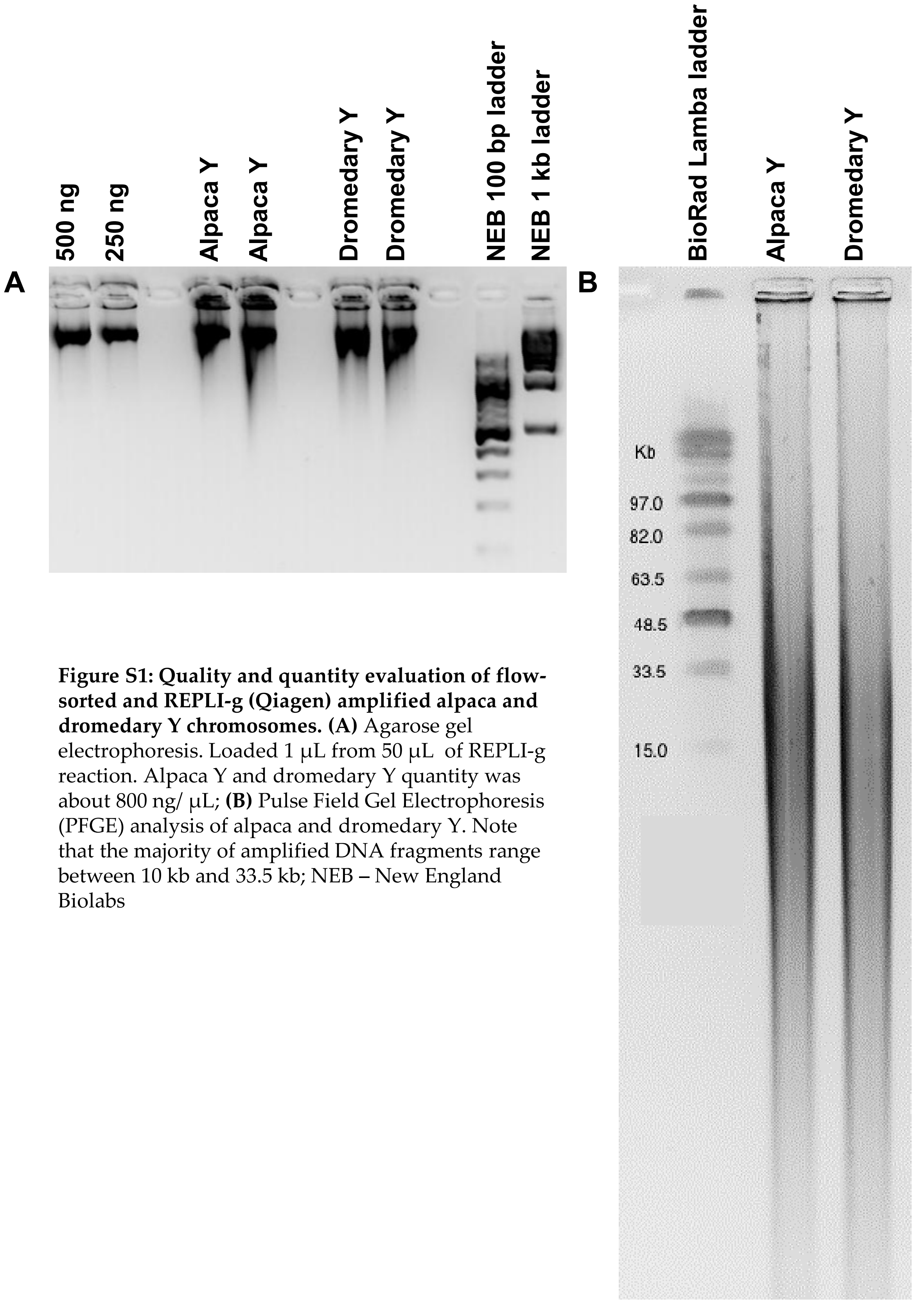

Figure S1: Quality and quantity evaluation of flow-sorted and REPLI-g (Qiagen) amplified alpaca and dromedary Y chromosomes. (A) Agarose gel electrophoresis. Loaded 1 μL from 50 μL of REPLI-g reaction. Alpaca Y and dromedary Y quantity was about 800 ng/ μL; **(B)** Pulse Field Gel Electrophoresis (PFGE) analysis of alpaca and dromedary Y. Note that the majority of amplified DNA fragments range between 10 kb and 33.5 kb; NEB – New England Biolabs

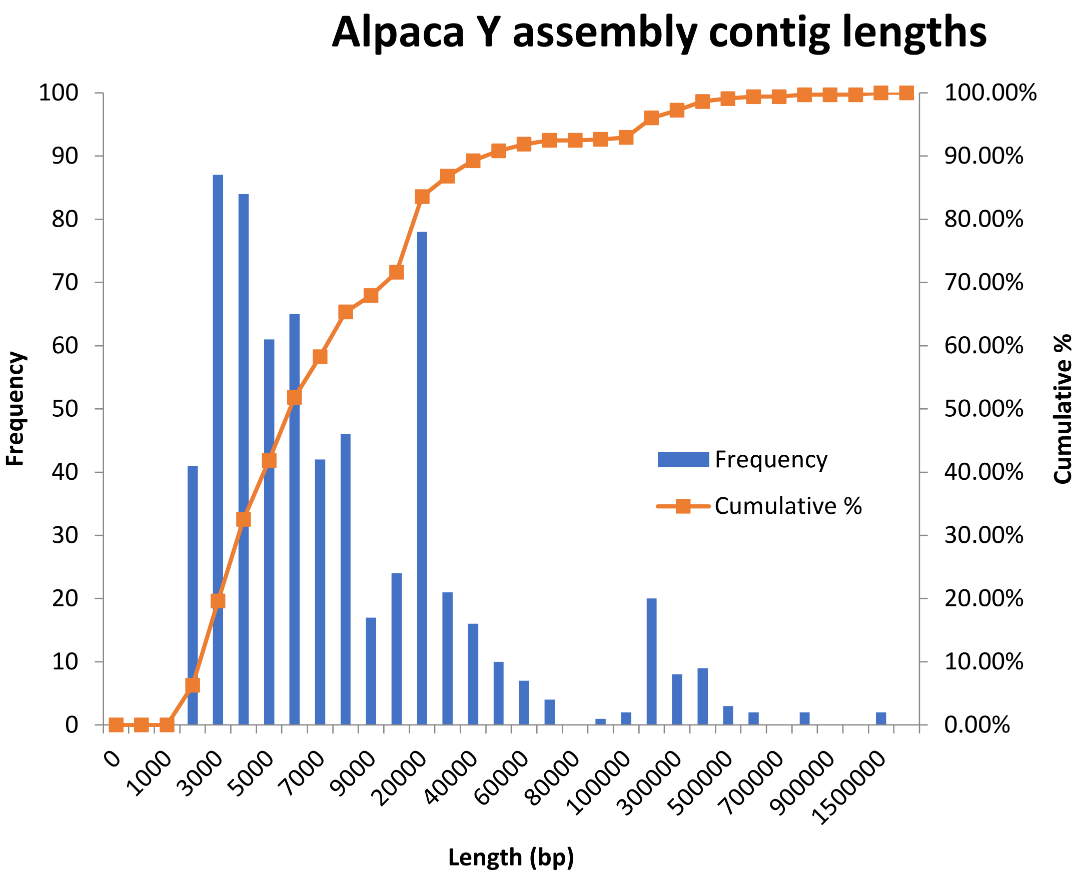

Figure S2. Length distribution of the 652 contigs assembled from long and short reads of the alpaca flow sorted Y chromosome. The x-axis shows the length in base pairs; the y-axis shows the number of reads of a certain length, and the z-axis shows the cumulative percentage of reads of a particular size or shorter denoted by the orange line.

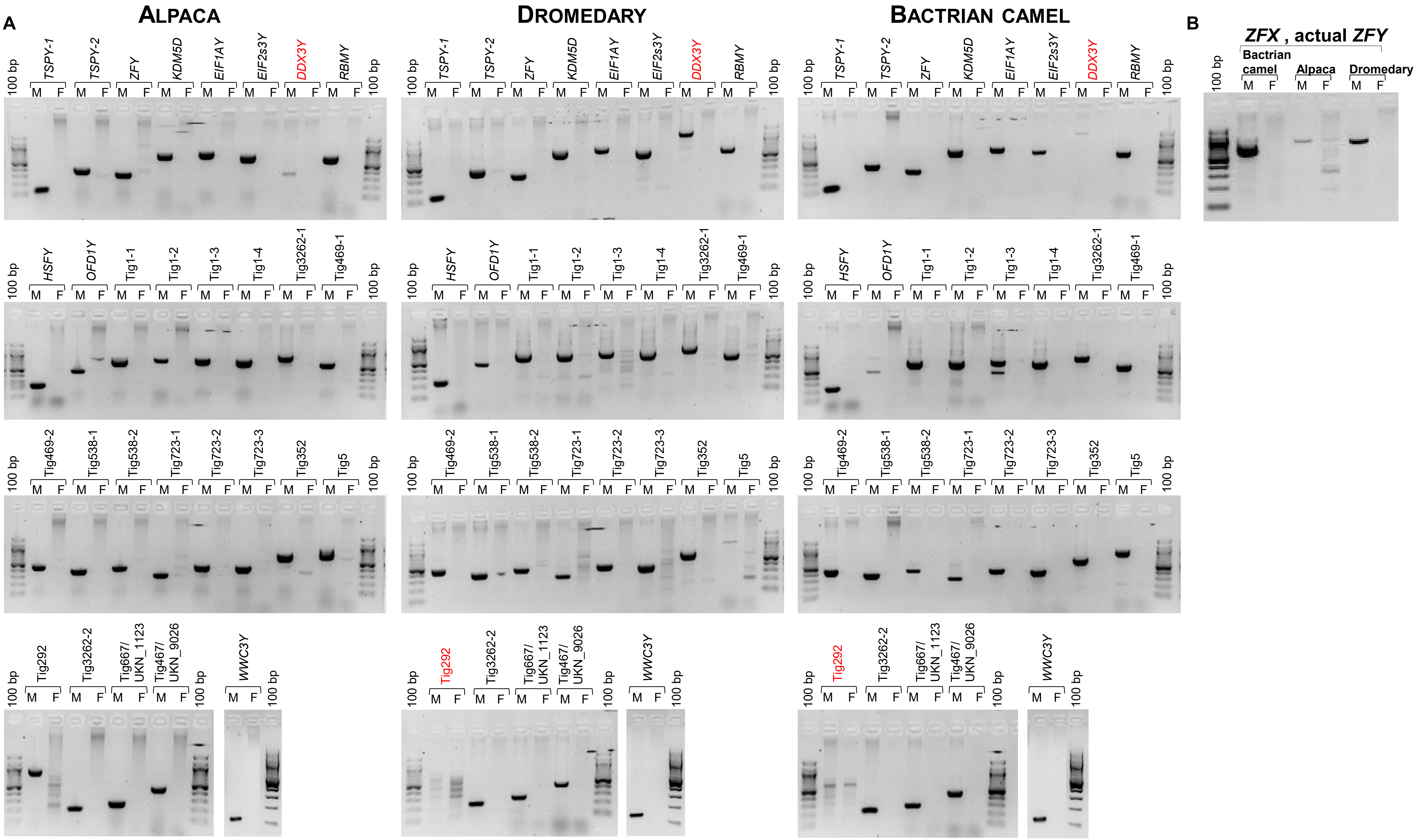

Figures S3. (A) Images of agarose gel electrophoresis showing PCR results with primers designed from 18 MSY contigs and/or 12 specific genes (see Table S1 for primer sequences) in male (M) and female (F) gDNA of the alpaca (left), dromedary (middle) and Bactrian camel (right). The majority of primers produced male-specific PCR products of the expected size in all three species, thus confirming their MSY origin, correctness of the assembly and evolutionary conservation. The exceptions are shown in red font: primers for *DDX3Y* amplified different size products in alpaca and the two camels, and neither of these corresponded to the expected size (Table S1), though the amplification was male-specific in all three species; Primers for contig Tig292 did not amplify in the dromedary and Bactrian camel. **(B)** PCR results with primers designed from Bactrian camel sequence XM_032475351.1 denoted as *ZFX*. The primers show male-specific amplification in all three camelid species and suggest that this sequence corresponds to *ZFY*. 100 bp: molecular ladder by New England Biolabs.

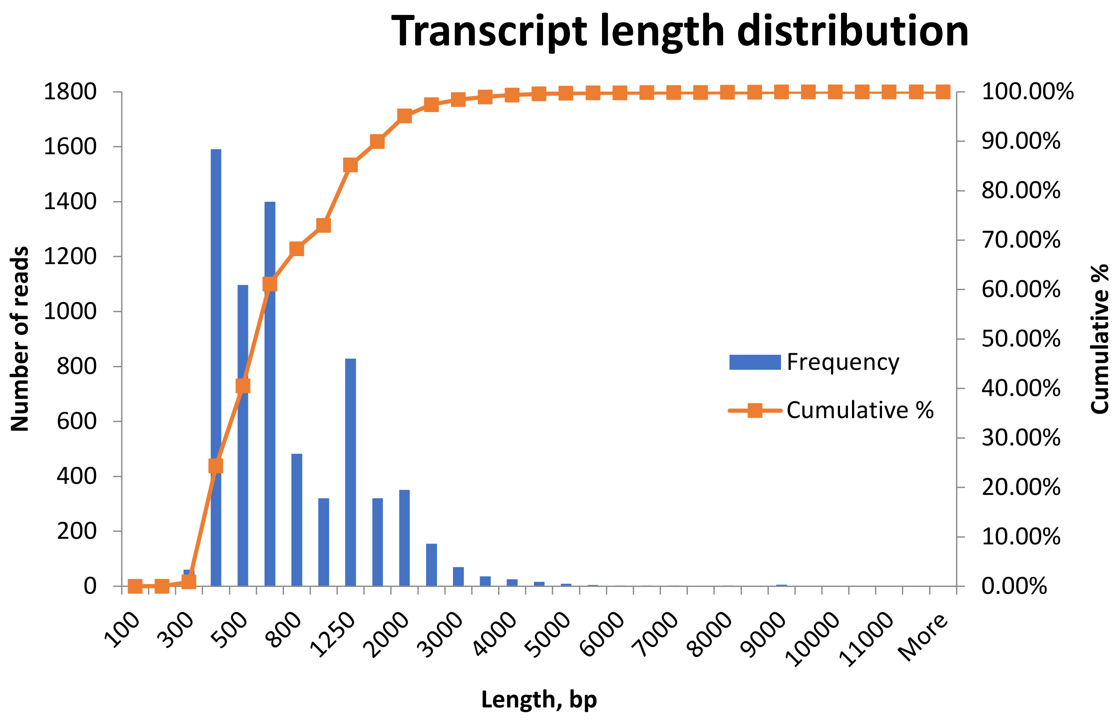

Figure S4. Length distribution of assembled transcripts with BLASTp results. The x-axis shows the length in base pairs, the y-axis shows the number of reads of a certain length, and the z-axis shows the cumulative percentage of reads of a particular size or shorter, denoted by the orange line.

…continued

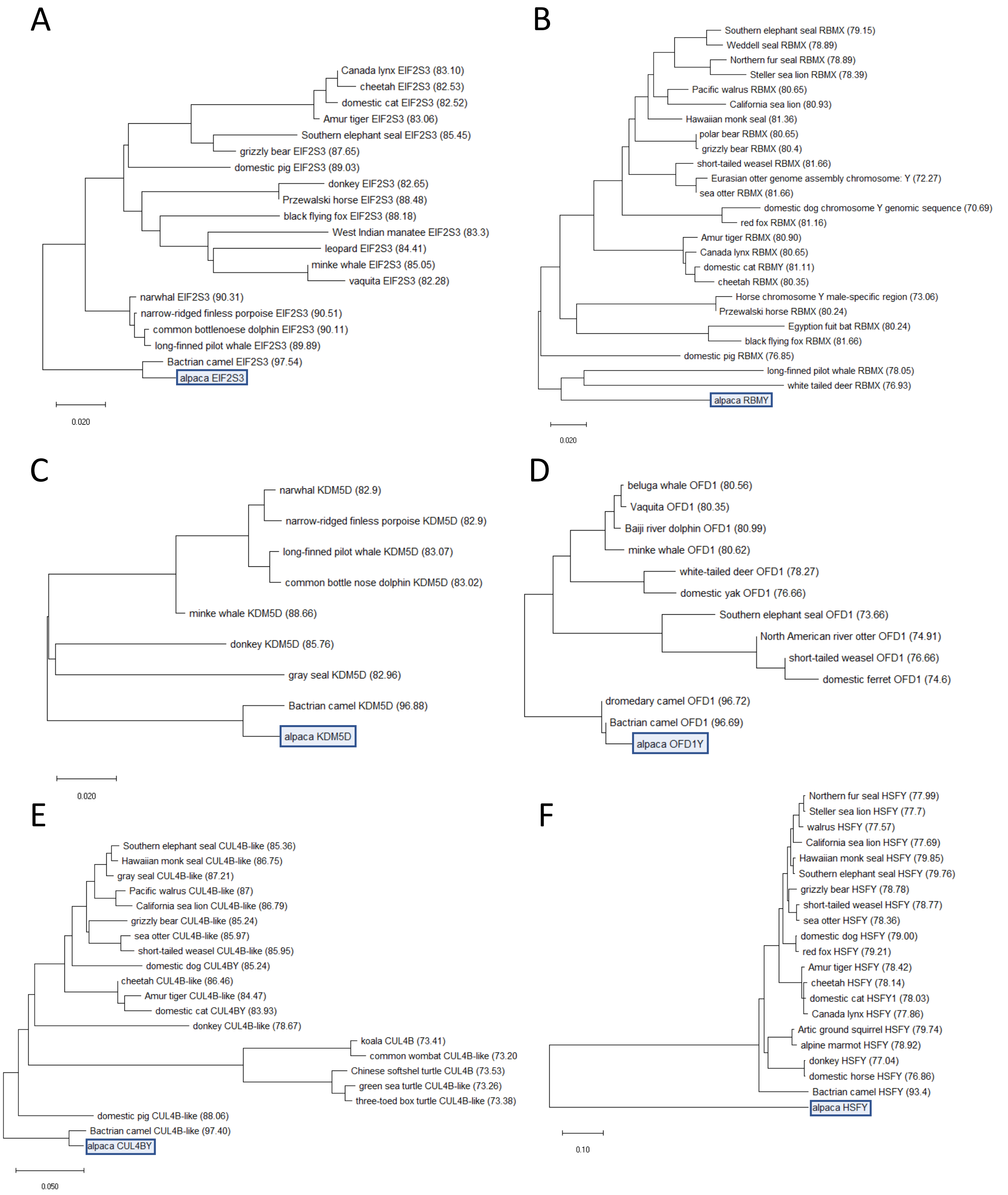

Figure S5A. Neighbor-joining trees showing the closest related sequences to alpaca X-degenerate genes in Genbank as determined by discontiguous megaBLAST: (**A**) *EF2S3AY*; (**B**) *RBMY*; (**C**) *KDM5D*; (**D**) *OFD1Y*; (**E**) *CUL4BY*; (**F**) *HSFY*. All trees are mid rooted with the branch corresponding to alpaca. Branch length is equivalent to relative distance between two sequences.

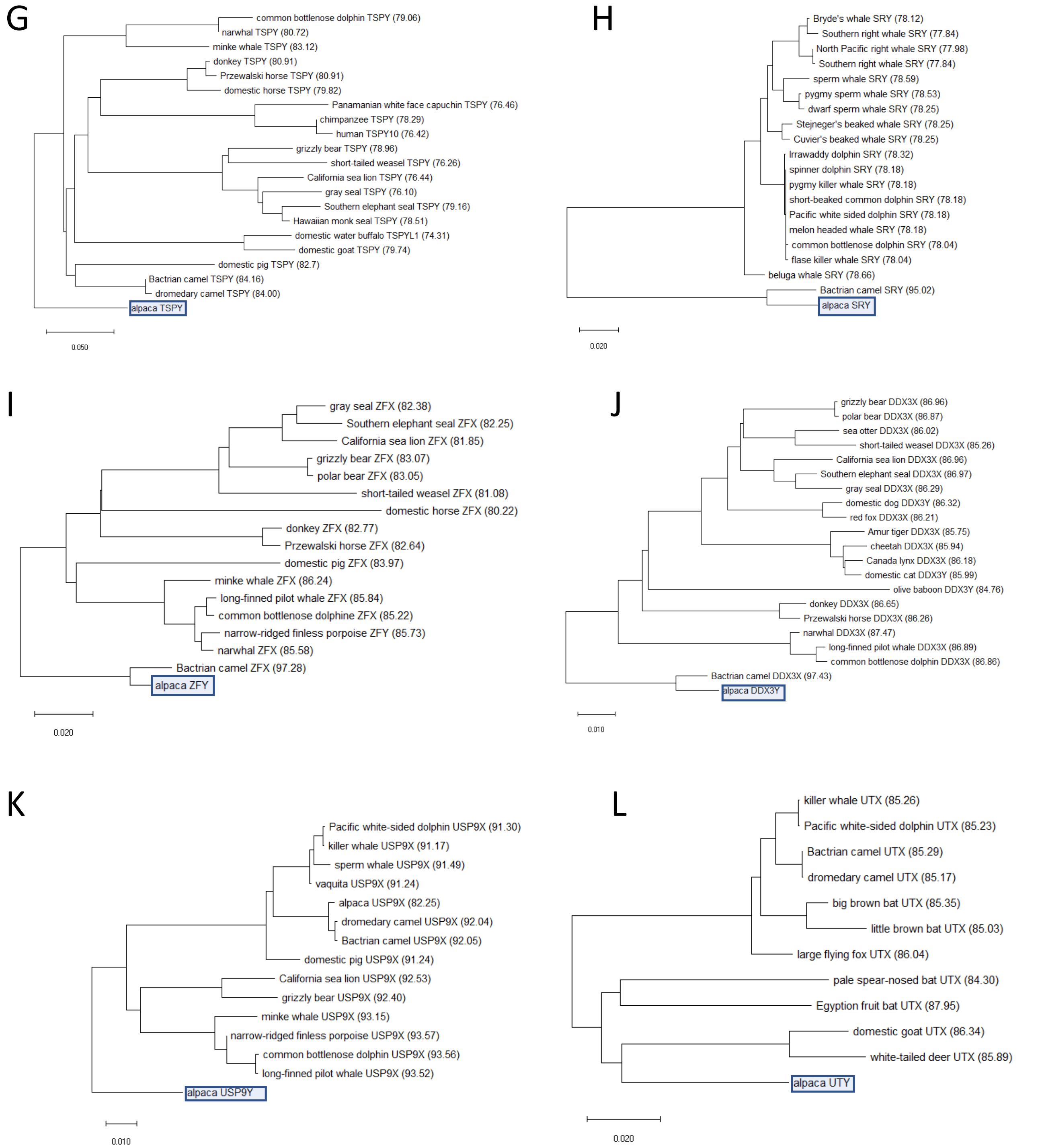

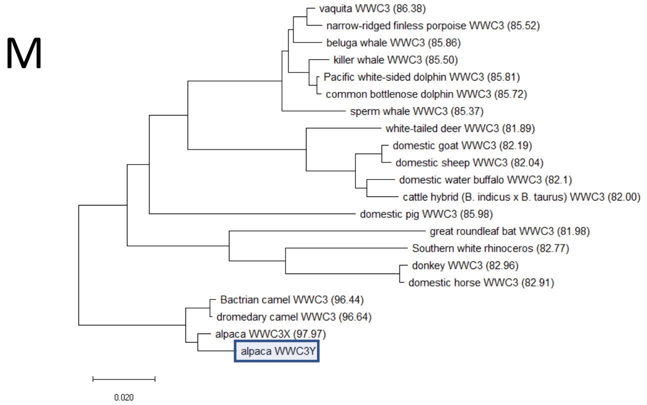

Figure S5B. Neighbor-joining trees showing the closest related sequences to alpaca X-degenerate genes in Genbank as determined by discontiguous megaBLAST: (**G**) *TSPY*; (**H**) *SRY*; (**I**) *ZFY*; (**J**) *DDX3Y*; (**K**) *USP9Y*; (**L**) *UTY*, and (**M**) *WWC3Y*. All trees are mid rooted with the branch corresponding to alpaca. Branch length is equivalent to relative distance between two sequences.
